# Risk Profiling from the European Statistics on Accidents at Work (ESAW) Accidents′ Databases: A Case Study in Construction Sites

**DOI:** 10.3390/ijerph16234748

**Published:** 2019-11-27

**Authors:** Mara Lombardi, Mario Fargnoli, Giuseppe Parise

**Affiliations:** Department of Chemical Engineering Materials Environment (DICMA), Sapienza-University of Rome, via Eudossiana 18, 00184 Rome, Italy; mara.lombardi@uniroma1.it (M.L.); giuseppe.parise@uniroma1.it (G.P.)

**Keywords:** occupational health and safety, accident databases, ESAW variables, construction, electric risk, cluster analysis, risk profiling, safety management, cindynic approach

## Abstract

The number of accidents and victims in the construction sector has not decreased significantly despite the increasingly stricter laws and regulations. The analysis of accidents, as well as their root causes and determinants can certainly contribute to the development of more effective preventive interventions. The present study proposes a methodology for the analysis and synthesis of data provided by accidents statistics with the goal of defining specific risk profiles based on the accidents determinants, their variables, and how they interact with one another in influencing the occurrence of an accident. For this purpose, a procedure capable of extracting this type of information from the European Statistics on Accidents at Work (ESAW) database was developed. In particular, data processing and aggregation are performed by means of the synergic use of the Matrix of Descriptors (MoD) and cluster analysis. To validate such a procedure, the analysis of fatalities due to electrical shocks was carried out. The results achieved allowed us to elicit valuable information for both safety managers and decision makers. The proposed methodology can facilitate a systemic analysis of accidents databases reducing the difficulties in managing reports and accident statistics.

## 1. Introduction

The high rate of occupational accidents in the construction industry represents a major concern in many countries (as outlined by numerous studies and statistics, e.g., in [1,2,3,4,5,6,7]), despite the efforts made by governments and public bodies to reduce it [8,9,10,11,12]. One of the main issues to deal with such a phenomenon consists in performing a proper occupational risk assessment (ORA) [13] in order to provide effective safety management measures since the project level [14,15,16]. However, the analysis of the accident causality and the definition of the related safety measures represent a complex task [17,18,19]. Traditional tools for occupational safety management at the engineer′s disposal are based on the legislation requirements, technical standards, safety guidelines, investigation reports and accident statistics [20]. The analysis of the latter can provide essential information to designers, project and safety managers for the implementation of adequate preventive measures [21,22,23]. In literature, numerous studies fostering such a cue discuss the quality of data provided by official accidents reports [24,25]. In particular, with reference to the European Statistics on Accidents at Work (ESAW) system of the European Union (EU), Molinero-Ruiz et al. [26] analyzed the reliability and validity of the ESAW variables′ coding system. They argued that further studies are needed to augment the quality of this database as it represents the basis for decision-making aimed at improving occupational safety. Salguero-Caparros et al. [27] reviewed numerous empirical studies on the way investigation reports on occupational accidents are carried out, considering the usability of ESAW variables to support the definition of preventive measures. This study brought to light the difficulties of inspectors in providing information regarding accidents adequately, consequently reducing the effectiveness of this type of databases. The problems related to accidents reporting were highlighted also by Schenk and Öberg [28], who stated that some difficulties with identifying chemical accidents even when the coder is guided by an ESAW compatible reporting form. Similarly, other studies have reported the limitations of ESAW system in providing an accident scenario in the agricultural field [29], analyzing the associated accident reports to reduce such flaws [30]. Palamara et al. [31] proposed the joint use of the Self Organizing Map (SOM) artificial neural network as a supportive tool for the k-means clustering algorithm to filter ESAW data on accidents occurred in the wood processing industry. An augmentation of this approach to deal with accidents′ variables more effectively was proposed by Comberti et al. [32], who also provided a review of studies of ESAW data treatment models to support risk assessment.

Considering the construction sector, Carrillo-Castrillo et al. [33] examined the accident investigation by public authorities and how this affects the identification and prioritization of preventive activities. Reviewing the accident models proposed in literature, the Authors emphasized the lack of specific studies making the usability of accidents′ statistics variables and coding, proposing a procedure based on ESAW variables that aims at establishing a correlation between accident causation and workers′ characteristics. On the one hand, the results achieved show how human factors can influence the accident causes identified in official investigations. On the other hand, this study also highlights the problem of identifying multiple factors that influence the mode of occurrence of an event positively or negatively (i.e., the so called “modulators”). 

Moreover, the quality of the information extracted from accidents reports is also based on the ability of the analysts in using them [34], and more research needs to be carried out to deal with this potential bias [35,36]. Some methods for accidents classification and analysis do not rely on the same taxonomy of contributing factors, allowing the analysts a certain degree of freedom that reduces the reliability of the results [37]. As noticed by Hola et al. [38], the analysis of accidents should be carried out in a standardized manner in order to allow the comparison of results of different studies. For these reasons, they proposed a methodology for the classification of the causes of occupational accidents involving construction scaffolding into generic groups that include technical, organizational, and human causes. In this way, the information collected from the database can be used for prioritizing and developing preventive measures. 

Such a “predictive” role played by the analyses of accidents databases is referred to the capability of exploring accident patterns to put forward recommendations for accident prevention [39,40,41,42]. In fact, the analysis of accidents can support safety engineers in better understanding the factors generating them, providing useful information on the characteristics of some recurring critical elements, especially those induced by errors and/or procedural omissions [43]. Ayhan and Tokdemir [44] have recently provided a literature review of studies focusing on the extraction of information on accidents based on databases, as they represent a fundamental means for determining accidents′ causes as well as developing better safety systems and preventive measures. Consequently, they developed a methodology for incident analysis (i.e., considering both accidents and near-misses) based on data collected at the construction site. On the one hand, such an approach resulted in being more effective than previous studies as it is based on real data collected at various construction sites. On the other hand, the quality of the output depends on the experience of the analysts and data collection activities can be very time- and resource-consuming. Alternatively, several Authors have suggested the use of compensation authorities′ databases for gathering information on accidents to be used to depict safety indicators [45,46,47]. 

However, as recently observed by Love et al. [48], the use of this type of data might lead to an underestimation of the phenomena, consequently reducing the quality of the captured information. In fact, these types of analyses provide indications at a general level, which need further processing to take into account the complexity arising from the combination of multiple factors that can lead to an accident [23]. In such a context, the cindynic approach [49] addressing the analysis of systemic sources and drivers of risk should be applied to extract more consistent information. 

Based on these considerations, it is deemed that the use of accidents statistics represents one of the most powerful approaches to capture information able to improve safety issues in construction projects. However, difficulties in dealing with these data properly have emerged, shedding light on the need of further studies on tools capable of providing practical and specific results efficiently.

The present study aims at contributing to the latter issue, proposing a methodology based on the k-means cluster analysis [50,51] for eliciting safety information from ESAW accidents reports in a systemic manner. Such a tool is largely used in exploratory data analysis due to its simplicity and easiness of use [52]. It allows the analysts to identify groups of similar objects (clusters) from a sample population: in particular, the method uses a centroid-based approach to minimize within-cluster variations, while different clusters have to differ from one another to the highest degree possible [53]. Accordingly, k-means clustering can allows the individuation of clusters of accidents with common characteristics.

More in detail, the accidents reports considered in this study are the ones provided by the database Infor.MO [54] by the Italian Compensation Authority (INAIL), where occupational fatalities and accidents leading to serious injuries are registered and classified. Differently from the general database on occupational accidents, Infor.MO is a database based on the ESAW latest protocol that makes additional information on the registered accidents available. In fact, it provides a brief description of the accident, a record card showing the details of the injury factor, as well as the determinant or the determinants when one or more factors have contributed to the accident′s occurrence [55]. The use of ESAW databases can allow the reduction of the drawbacks related to the limited information of traditional statistics on occupational accidents [23]. Actually, this permits the definition of a set of accidents descriptors as a base for further characterization analysis of the data considering multiple variables. In this ambit, clustering tools can lead to a stratification of the results providing useful information on the relationship between the accidents occurred in a specific sector and the related descriptors, i.e., the variables of integration characterizing the phenomenon. For this purpose, based on a systemic classification of these accident descriptors, data collected are translated into Boolean variables and then analyzed through a k-means cluster analysis [56,57]. 

## 2. Materials and Methods 

### 2.1. The Infor.MO Database

As mentioned earlier, the database Infor.MO [54] provides not only traditional statistics on the number of occupational accidents, but also additional data describing each accident. As observed by Lombardi and Rossi [56], the information that can be collected by the Infor.MO database consists in the following data:

1. Type of accident: e.g., fatality, serious injury, or disabling accident.
2. Data related to the event: e.g., date, hour, no. of people involved, type of working activity carried out when the accident occurred, type of company, economic sector of the company, etc.
3. Description of the accident: a synthetic description of the accident is provided based on the reports of the authorities.
4. Type of energy transfer: energy exchange, energy release, and improper use of energy.
5. ESAW variables: following the ESAW rules, data such as deviation, material agent, contact type, etc. are codified.
6. Information on the victim: age, sex, nationality, working experience, type of lesion/injury, part of the body injured, etc.

**Figure 1:**
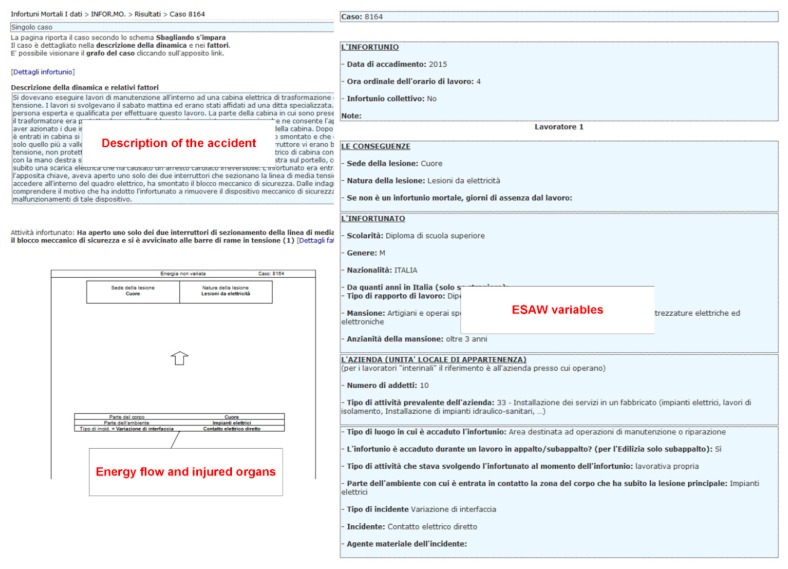
Example of the information provided by the Infor.MO database (adapted from [58]).

In Figure 1 data sheets extracted from the Infor.MO database regarding a fatal accident in the construction sector are shown [58]. 

### 2.2. Cluster Analysis for Occupational Safety

Cluster analysis is a well-known statistical method for classification [59], grouping together objects whose patterns of scores on certain variables are similar [60]. In literature, numerous applications of cluster analysis tools in the field of occupational safety can be found, which are aimed at putting forward predictive information on safety issues [61]. For instance, Arocena and Nunez [62] used the Euclidean distance between vectors of the standardized values of 12 variables in order to classify Occupational Health and Safety (OHS) management systems among manufacturing companies, providing a relationship between the different types of the classified OHS systems and the number of accidents. Similarly, Champoux and Brun [63] applied a hierarchical cluster analysis to describe the employers′ representations of OHS safety problems and hindrances to improve prevention within the company.

In addition, cluster analysis was also used to extract and categorize data from statistics: in fact, several studies focused on the analysis of specific risks based on statistics concerning incidents. For example, Engkvist et al. [64] applied the cluster analysis for the statistical treatment of data collected among nursing personnel in order to bring to light the relationship between the risk of back injuries and the risk factors or protective factors associated to these working activities. Differently, Raviv et. al. [65] applied cluster analysis to statistics on near-misses in the use of cranes in the construction sector. More in detail, this research project was articulated into three different main phases, concerning: data collection; cluster analysis; and the identification of the incident′s total risk potential by means of the Analytic Hierarchy Process (AHP) method. As already underlined, other studies applied the Kohonen′s Self-Organizing Map (SOM) and the k-means clustering algorithm to identify the most critical groups of occupational accidents from ESAW data [31,32]. Therefore, on the one hand, the benefits emerging from the use of cluster analysis and its extensions for the elicitation of safety information from incidents statistics to be used also in a predictive manner are clearly deemed. On the other hand, although the remarkable results achieved by these studies, their usability at a practical level appears limited due to the difficulties that might arise in data collection and their proper manipulation to establish a relationship between them and the working activities.

### 2.3. Systemic Approach

**Figure 2:**
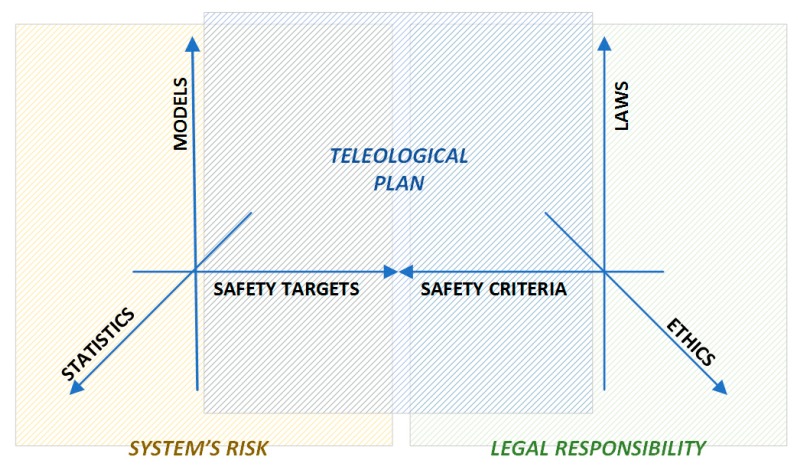
The cindynic hyperspace representation (adapted from [67]).

As mentioned above, when dealing with critical factors resulting from complex activities a systemic approach for risk analysis is needed. In this ambit, the theoretical framework provided by the cindynic theory [66] foresees the synergy between statistics (data), modelling (methods), goals (finalities), rules (laws), and values in the so-called “cindynic hyperspace” (Figure 2).

In detail, on the one side, the combination between statistics and modelling (the epistemic-mnesic space) allows the definition of the characteristics of the system′s risk profile (safety targets). On the other side, a regulatory action supported by ethical criteria (the ethical-axiological space) defines the legal responsibility profile by setting safety criteria. Between these two spaces, the teleological plan can be identified, allowing the control of the compliance through the comparison between the acceptability criteria (i.e., safety criteria) and safety targets (i.e., mandatory requirements), providing information on the thorough profile of risks. Such a holistic representation of the level of danger highlights the role of the knowledge that can be acquired by accidents statistics on the proper definition of a risk profile (epistemic-mnesic dimensions), while the behavior of operators and supervisors is characterized by the ethical-axiological dimensions [67].

The translation of such an approach in the practical analysis of an accident can be carried out by means of the Reason′s Swiss Cheese Model (SCM), according to which an accident can be ascertained to one or more of four failure domains: organizational factors, supervision, preconditions and specific acts [68]. In other words, when the holes of the SCM defensive layers (representing technical, operational, and organizational barriers) are lined up, an existing hazard can cause an accident [69]. 

Therefore, the above mentioned domains need to be represented by accident descriptors tailored on the main areas of relevance used to describe the occurrence of an accident in official reports. For this purpose, the following four descriptors were selected following the accident report schemes provided by the International Labour Office (ILO) guideline on the official reporting requirements of occupational accidents [70]: hazards (representing the hazards in the working environment); hazardous events (i.e., the contact—mode of injury); responsibilities (describing the responsibilities of the worker/entrepreneur in both the cold phase (i.e., planning) and in the hot phase (i.e., during the working activity); and compliance (indicating whether, when the accident occurred, the compliance with safety requirements was fulfilled or not). To summarize, the analysis of the accident descriptors allows the evaluation of the failure domains providing information on risk sources and drivers, and consequently on which actions are needed to reduce the occurrence of the same type of accident.

### 2.4. Research Approach

**Figure 3:**
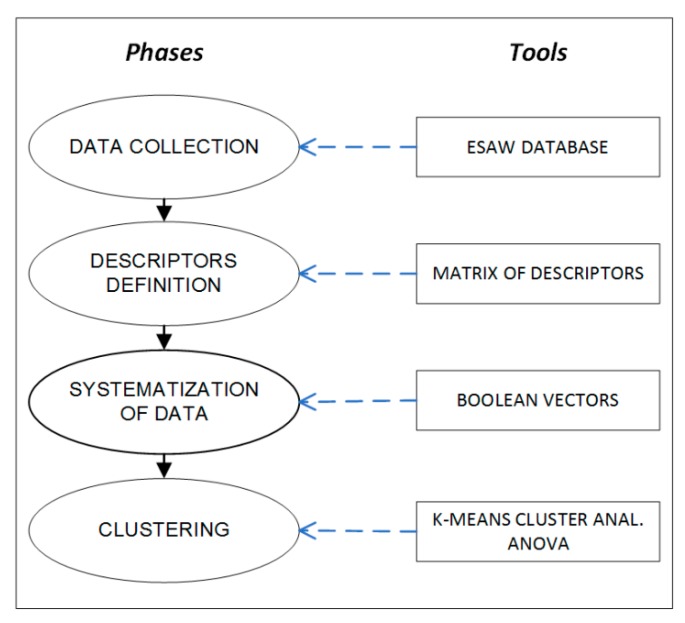
Scheme of the proposed methodology.

**Figure 4:**
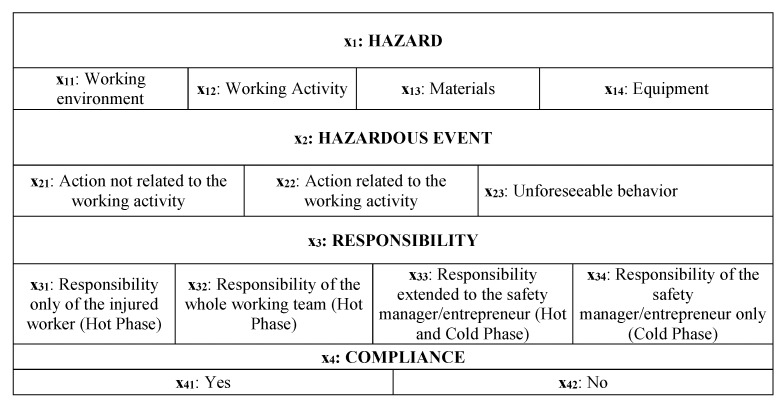
The Matrix of Descriptors (MoD) framework.

**Figure 5:**
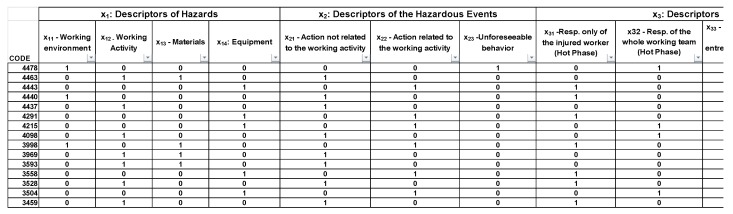
Excerpt of the MoD application (the MS Excel^®^ software was used).

**Figure 6:**
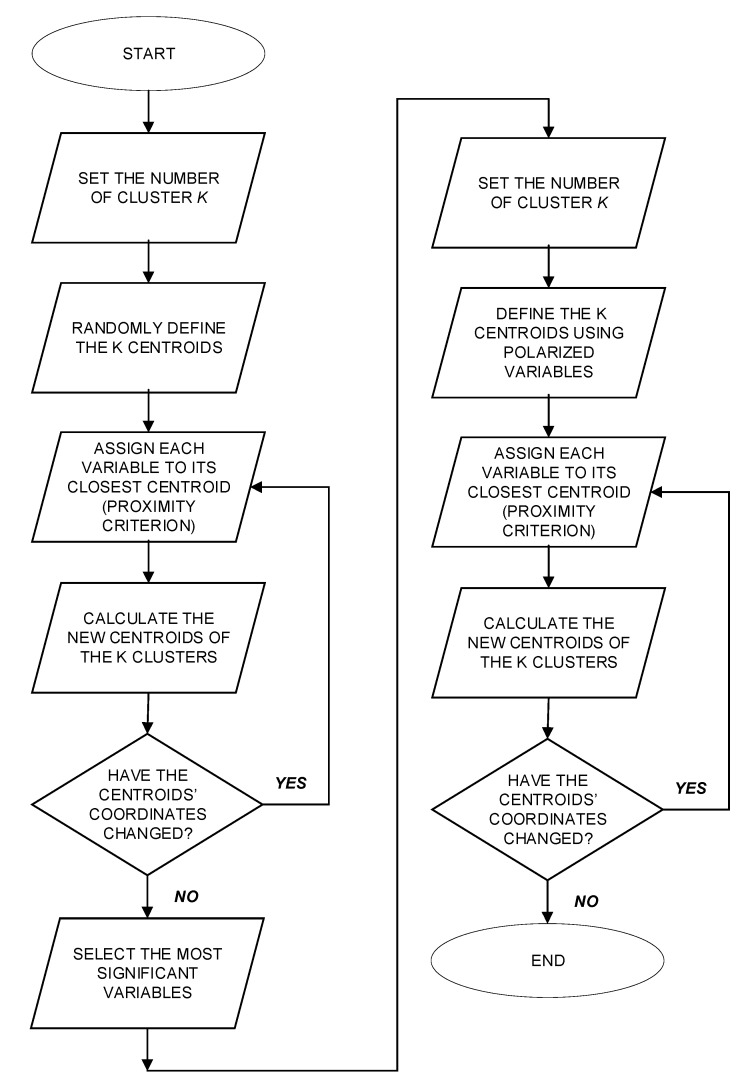
Scheme of the cluster analysis application.

Based on the above considerations, the aim of the present study consists in providing a methodology tailored for using data from already existing databases, such as Infor.MO, which is capable of providing codified information to build up a set of variables describing the way an accident occurs. More in detail, such a methodology, summarized in Figure 3, consists of the following main phases:

1. Data collection: data related to a specific type of accidents (e.g., accidents due to electric shock in the construction sector) are extracted from the Infor.MO database.
2. Identification of the descriptive variables of accidents: information provided in each accident report is analyzed in order to depict the sub-descriptors of the system, which are translated into the *n* variables into the related *k* types of the reference areas (i.e., the descriptors). In practice, the general scheme of such a classification consists in relating the four different types of descriptors (hazard, hazardous event, responsibility, and compliance) with the x_i_ variables, where *i* = 1, … 4 (maximum number of areas of relevance) indicates the number of the descriptors, while *j* = 1, … *m* represents the number of the different sub-descriptors for each descriptor. The output of such a process consists in the definition of the “Matrix of Descriptors” (MoD) depicted in Figure 4, where each descriptor is composed by different sub-descriptors, representing the descriptive variables of the system that can be extracted from the ESAW accident reports. It has to be noted that in this way the logical disjunction of the x_ij_ variables is guaranteed. In total, 13 variables were identified (*n* = 13).
  The Matrix of Descriptors allows us to merge the characteristics of the epistemic-mnesic and ethical-axiological spaces of the cindynic approach, representing a tool aimed at “filtering” accidents data in order to elicit their main features in terms of safety targets and safety criteria. Accordingly, the selection of descriptors and sub-descriptors was made taking into account both the system′s risk features and legal responsibilities issues related to an accident, consistently with the variables suggested by the ESAW system and the ILO guidelines [70].
3. Systematization of data extracted from the accidents database by means of Boolean coordinates: categorical information is translated into dichotomous variables. In other words, the x_ij_ variables that describe an accident are translated into an algebraic vector by means of the Boolean n-tuple of coordinates in the space R^n^. For this purpose the MoD is used, filling it with Boolean values (i.e., “0” when the accident is not affected by a certain variable; or “1” when the accident is affected by a certain variable). In Figure 5 an example of the MoD application is shown (the code number used is the one reported in the Infor.MO database).
4. Cluster analysis application aimed at identifying homogenous groups of accident cases based on the x_ij_ variables systematized in the previous phase. In other words, the set of observations is represented by the algebraic vectors defined above (corresponding to the *n* variables) with the goal of partitioning them into *k* (≤ *n*) sets (i.e., the clusters), where the algebraic vectors are assigned to a specific cluster following the criterion of “proximity” to the initial centroid. Based on this, in our context the use of the k-means clustering approach [59,60] is foreseen twice (Figure 6): the first time the application is aimed at defining the most relevant variables characterizing the type of accidents analyzed (which we called “polarized” variables), while the second application is focused on verifying the significance of this output, refining the mutual relationships among the variables to better understand the accident scenario. More in detail, the first series of iterations is carried without assigning the centroids in advance: the coordinates of centroids are randomly assigned by the software (in this study the IBM SPSS^®^ version 5.0 software (Armonk, NY, USA) [71] was used). The results obtained allows the definition of most relevant cluster solutions and the related coordinates of the centroids. These coordinates are used for further iterations, which end when the new centroids′ coordinates do not change [53]. The validation of the results is carried out by means of the Analysis of Variance (ANOVA) test [72]. The result of this first clustering process consists in the individuation of the most relevant variables, i.e., those representing the most impacting accidents′ determinants (the “polarized” variables). Afterwards, as illustrated in Figure 6, the whole procedure is repeated using these “polarized” variables as the input coordinates of initial k centroids. For this purpose, a new transformation into dichotomous variables (the value “1” is assigned to the “polarized” variables, while “0” is assigned to the other variables) was carried out to initialize the second clustering process.

Such an approach allows us both to verify the relevance of the selected variables, as well as to better evaluate the relationships among the different variables and how they interact with one another in influencing the occurrence of an accident (e.g., which is the most probable combination of determinants leading to an accident). It has to be noted that to select the maximum number *z* of clusters to analyze, in this study the criterion *z* = $\sqrt{n/2}$ is used and then the obtained clusters are evaluated comparing the number of cases in each cluster [73]. 

## 3. Case Study

**Figure 7:**
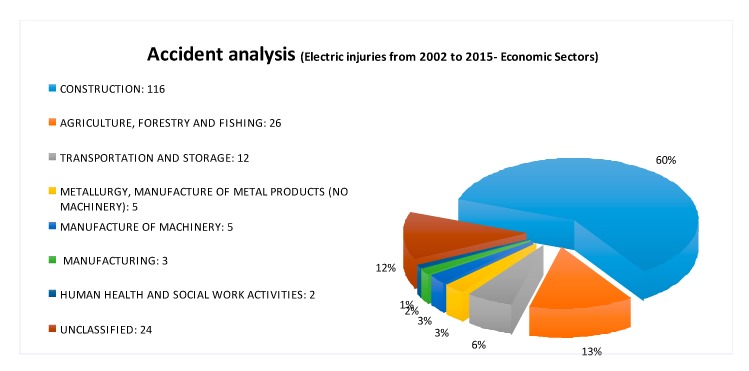
Occupational injuries due to electric shock in Italy (data source: [54]).

The proposed methodology was used to investigate the accidents due to electric shock in the construction sector, which occurred in Italy in the period 2002–2015. As mentioned earlier, the construction industry has been recognized as one of the most dangerous activities worldwide and the accidents involving electricity are of major concern especially in large construction sites [74,75,76]. Actually, the presence of workers belonging to different sub-contractors simultaneously, as well as the fact that most of them are not specifically trained and equipped against direct or indirect contacts with electrical parts make the occurrence of this type of accident in the construction industry higher than in the other sectors [77]. In Italy, analyzing data extracted from the Infor.MO database, the construction industry results in being the most affected sector by this type of accidents (Figure 7).

Based on the above considerations, the factors determining electrical accidents in construction sites are multiple and interwoven with one another. Therefore, filtering accidents data by means of their descriptors can shed light on these relationships, augmenting knowledge on their occurrence modes. Accordingly, following the procedure described in the previous section, the first step of the analysis consisted in collecting data related to electrical injury that occurred in the period 2002–2015 in the construction sector. A sample of 116 fatal accidents was extracted and analyzed to depict the characteristics of descriptors and sub-descriptors of each one of them. The results of this first analysis brought to light three main categories of accidents that can be elicited from the database:

A. The accidents occurred during generic activities, i.e., works where the use of specific personal protective equipment (PPE) against electric shock is not required. The injuries are due to the contact with high or medium voltage power lines.

B. The accidents occurred during maintenance activity of electrical equipment or devices. In these cases, the operator is usually skilled and trained for operating with electrical parts. Moreover, the use of specific PPE is foreseen.

C. The accidents occurred when dealing with machinery, equipment or devices that are not in compliance with mandatory safety requirements. In this case, the responsibility of the safety manager/entrepreneur in the “cold phase” is recognized.

**Figure 8:**
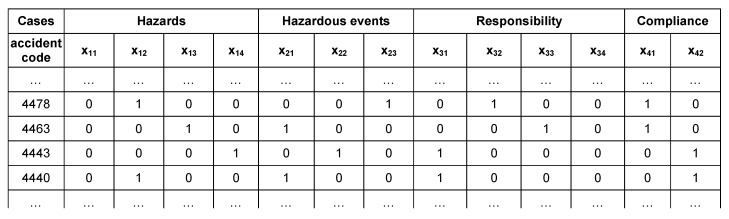
MoD application.

Moreover, it has to be noted that in the area of hazards, the variable x_11_ related to “working environment” refers to the “construction site”, while the variable x_13_ related to “materials” was explicated as “electricity distribution line”. Similarly, the area of the “Hazardous events” in this context refers to the “modes of contact” with the electricity line. The next step consisted in the systematization of data: as shown in the excerpt in Figure 8, for each case the accident′s variables were translated into an algebraic vector by means of Boolean coordinates. Among the 116 cases extracted from the database, 19 could not be used mainly due to the lack of some information. Consequently, 97 cases of fatal accidents were processed.

This transformation allowed us to apply the k-means cluster analysis through several iterations corroborated by ANOVA tests [72]. For this purpose, the IBM SPSS^®^ software was used (Quick Cluster procedure of IBM SPSS Statistics) [71], through which the statistical analysis of variables was performed (Table 1), as well as the condition of diagnostic features of the 13 dichotomous variables was verified to initialize the clustering (the Lance and Williams “distances matrix” was used). 

**Figure 9:**
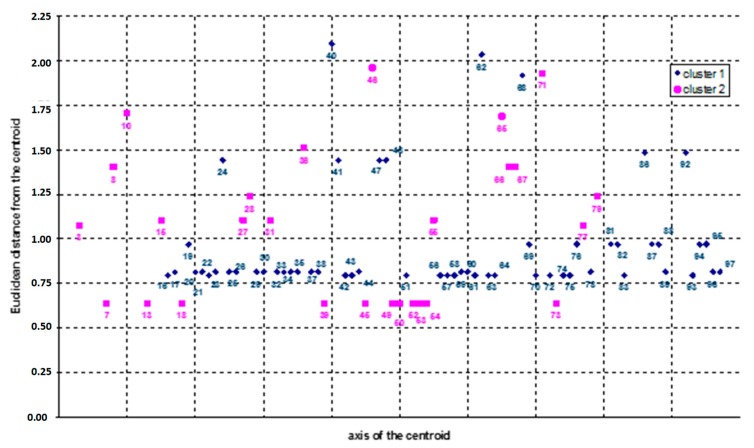
Distribution of the 2-clusters solution (Euclidean distance from the centroid axis).

**Figure 10:**
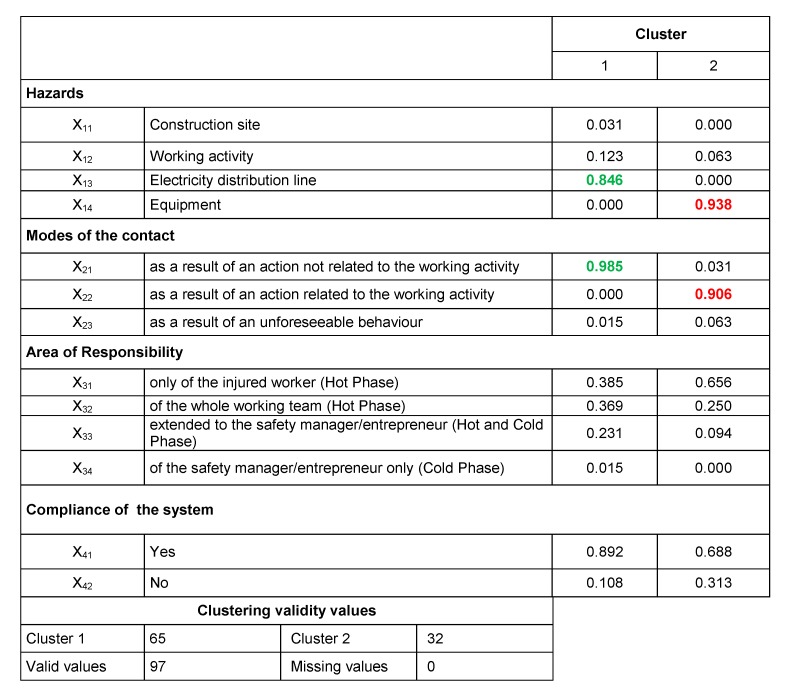
Results of the 2 clusters solution (values in red and green indicate the most relevant variables of each cluster).

Then, a solution including 2 clusters without a centroid was selected in order to preliminarily screen the impact of the variables on the accidents′ determinants. In particular, the first cluster included the type A and B cases, while the second cluster contains data related to the type C cases. The output of this first iteration is shown in Figure 9 and Figure 10. In detail, in the former a comparison between the Euclidean distances from the centroid of the examined accidents is reported. More precisely, each point in the figure represents an accident case and the “x” axis represents the centroid axis. 

In the latter figure (Figure 9), the comparison between the values of each variable of the two clusters is provided. The results of this analysis revealed that the two clusters are disjointed since they are polarized on different variables: as in Figure 9, cluster 1 is polarized on the variables x_13_ and x_21_ (values in green); cluster 2 is polarized on the variables x_14_ and x_22_ (values in red).

**Figure 11:**
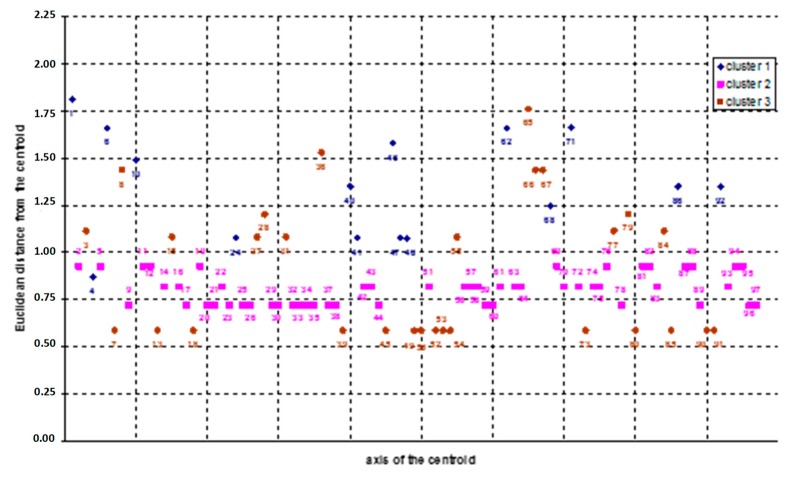
Distribution of the 3-clusters solution (Euclidean distance from the centroid axis).

**Figure 12:**
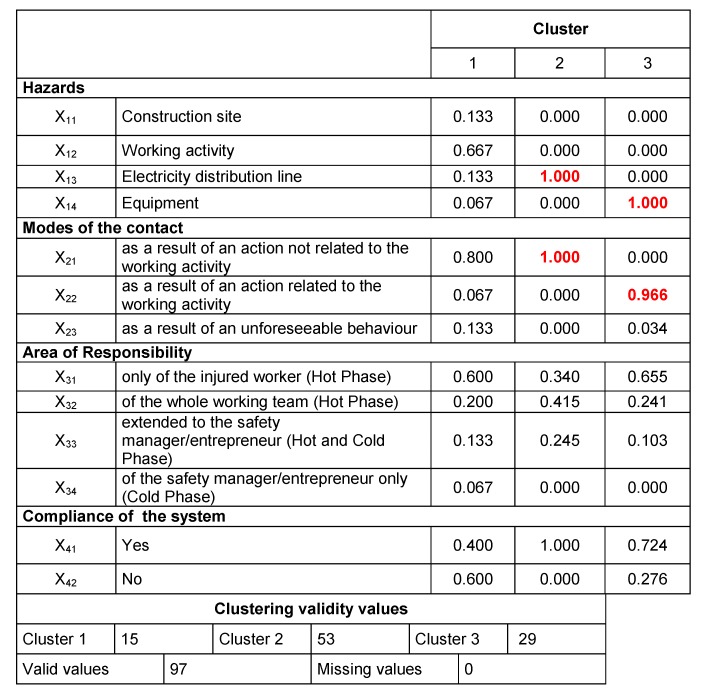
Variables′ values of the 3-clusters solution (values in red indicate the most relevant variables of each cluster).

As explained in the previous section, to select the maximum number *z* of clusters to analyze, in this study the criterion *z* = $\sqrt{n/2}$ was used [73]: i.e., since *n* = 97, the optimal number of clusters is 6 (*z* < 7). The results of the iterations related to the 3-clusters solutions are summarized in Figure 11 (showing the Euclidean distances from the centroid axis) and Figure 12 (reporting the variables′ values, where the most significant values are underlined in red).

**Figure 13:**
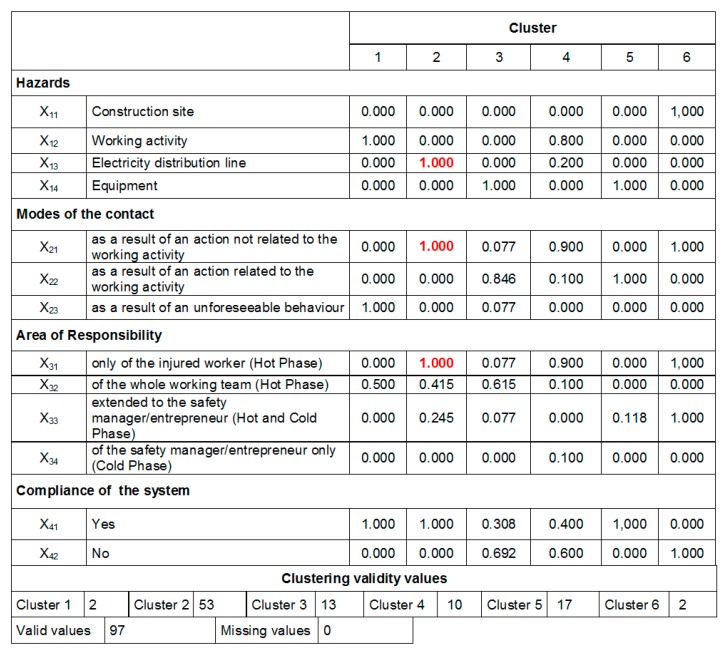
Variables′ values of the 6-clusters solution (values in red indicate the most relevant variables).

Similarly, in Figure 13 the output of the iterations related to the 6-clusters solutions is shown. These iterations revealed the relevance of cluster 2 (representing 53 cases), which resulted in being the same in both the latter solutions.

Afterwards, the obtained clusters were evaluated comparing the number of cases in each cluster [73]: in our case, a consistent number of cases was considered *n* > 10. Accordingly, with reference to the 6-clusters solution (Figure 13), cluster 1, 4 and 6 were considered not relevant.

Overall, reading through the numbers, the results obtained showed that most of accidents present the following characteristics: the contacts happened circumstantially, due to the interference between the working equipment/machinery used by the worker and the electric line (variables x_13_ and x_14_), both when carrying out an action not related to the specific working activity (variable x_21_) and when this action is related to the specific task of the worker (variable x_22_). These results were used as input for the second application of the k-means cluster analysis using the above-mentioned variables as “polarized” variables. 

**Figure 14:**
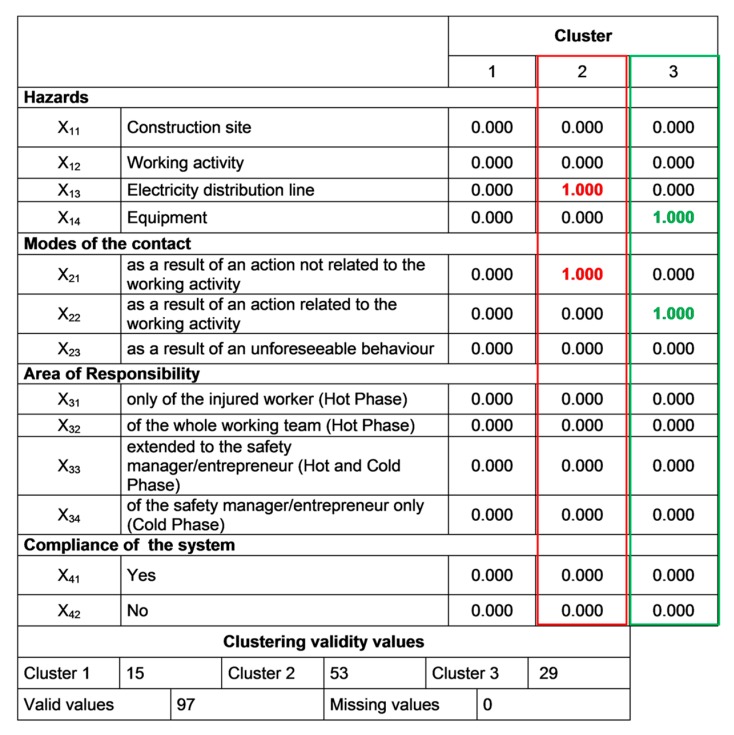
The 3-clusters matrix for the definition of the centroids (values in green and red indicate the most relevant variables of each cluster).

**Figure 15:**
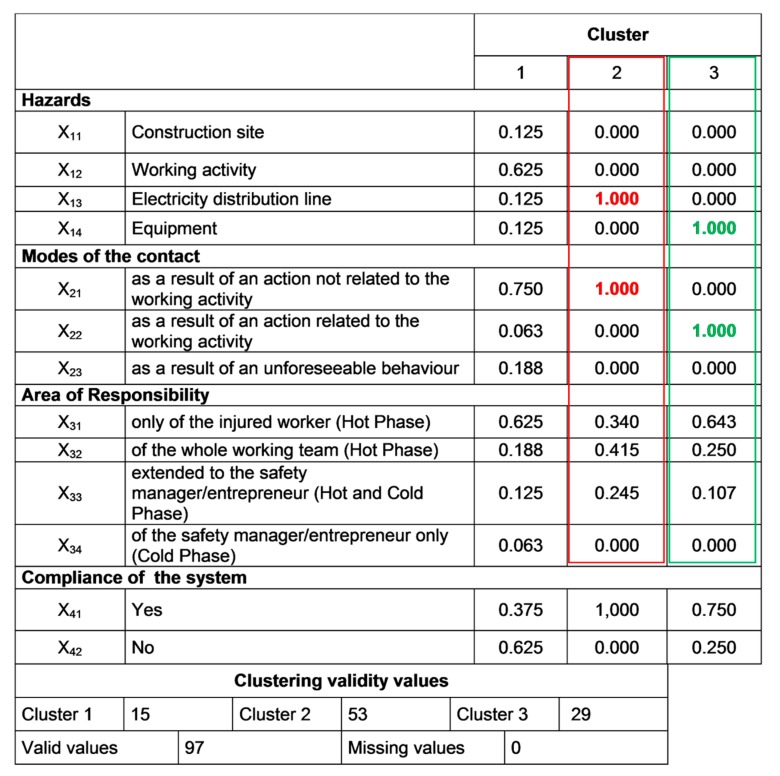
Results of the 3-clusters solution with centroids (values in green and red indicate the most relevant variables of each cluster).

Due to space limitations only the results concerning the 3-clusters solution are shown. Additional data related to the other iterations can be provided upon request to the authors. In detail, in Figure 14 the 3-clusters matrix used for the centroids′ setting is shown, while the final results of the 3-clusters solution are reported in Figure 15. 

Consistently with the output of the preliminary iterations, the final results shed light on the most influencing variables on the dynamics of the accidents: namely, x_13_ and x_21_ for cluster 2 (values in red); x_14_ and x_22_ for cluster 3 (values in green). Thus, with regard to the accident determinants in the work place, the electricity distribution line and working equipment are the most important variables. Instead, actions related to the working activity (cluster 3) and those not related the working activity (cluster 2) represent the mode of contact in the two risk profiles.

## 4. Discussion

### 4.1. Case Study Results

The results of data aggregation brought to light two main clusters (recurring patterns) both in 6- and 3-clusters iterations that consequently were considered consistent and representative of the phenomenon:

- Cluster 2, populated by 53 cases, which are characterized by direct contact with the electrical line during construction activities not related to electrical works. Namely, 42% of these accidents are due to a failure of the working team and/or the safety manager.
- Cluster 3, populated by 29 cases, determined by a failure of the worker (65%) when using a working equipment (e.g., a crane or a scaffold).

Although these two clusters are characterized by different accident modes, they present a common factor contributing to the occurrence of the fatalities, which is represented by the failure of humans (workers, co-workers, or safety managers). In fact, in both of them the human error plays a fundamental role, as in both risk profiles (cluster 2 and cluster 3) the mode of contact is characterized by foreseeable behaviors. Such a finding confirms the results obtained by other studies [74,75,78,79], underlining the fundamental role of human errors in safety management. 

Moreover, the use of working equipment as proximal cause of the accidents confirms the results of similar studies in the construction sector [80,81,82], stressing the need for more rigorous interventions also at the normative level as in the case of scaffolding [83]. The results obtained showed that multiple factors always influenced the accidents′ occurrence, confirming the need to take into account the interactions between the different aspects that characterize working operations. This is consistent with the research cues coming also from other fields, such as the agriculture [84] or the oil and gas sectors [85], confirming that the interdependencies among the different variables of an accident should be scrutinized as in most cases the determinants of a fatality are multiple and interconnected, overlapping with each other [36]. As far as the electrical accidents are concerned, the results show that most fatalities occurred to workers carrying out activities not related to specific electrical works (cluster 2). On the one hand, such an output is in line with the results of similar studies in other countries [75,85], highlighting the high rate of electrical accidents among construction workers different from electricians. On the other hand, this aspect fosters the need of a more accurate and multidisciplinary training of both workers and safety managers. In fact, according to the requirements of occupational health and safety legislation, OHS training of workers should concern the risks related to the specific activities and operations assigned to the worker. As a consequence, specific information and training (and protective equipment) on electric risks are provided only to those workers who perform electrical work (e.g., electricians), while other type of workers (e.g., painters or carpenters) hardly receive this specific training. Usually, safety managers (and the entrepreneurs) are likely to follow such a “rule of thumb”, underestimating the problem. However, as demonstrated in our case study, construction works are made of multiple activities where the presence of electricity and consequently of the electrical risks cannot be disregarded anymore, requiring a change both at the cultural and legislative levels. 

### 4.2. Research Implications

From a more general standpoint, the results of the study have demonstrated the capability of the proposed methodology in processing accidents data, generating useful information for the implementation of preventive and protective measures that are case-tailored to the specific working context. In fact, the analysis allowed us to define two main “risk profiles” (i.e., the ones deriving from cluster 2 and cluster 3 as in Figure 15). This type of output can provide predictions on accident patterns, showing at the same time safety flaws that need to be addressed to reduce the deviations that led to the accidents. Such a result certainly contributes to augmenting knowledge on the use of accidents data, providing a novel approach for filtering and aggregating ESAW data, which is not depending on the choices of the analysts. 

More in detail, as pointed out in the previous sections, numerous examples of tools aimed at extracting information from accidents statistics and data can be found in literature. However, most of them require a subjective intervention of the analyst and/or of a panel of experts [86,87]. Conversely, this study considered a database (Infor.MO) where the type of information made available is standardized. Hence, the effort made by the analysts in collecting information is reduced and consequently the potential bias in treating data is limited, in line with Hola et al. [38]. 

Moreover, unlike other studies, our approach is based on the analysis of reports related to a specific type of accident (electrical shock), including in the analysis also distal factors such as responsibilities in the cold phase (i.e., at project level) in accordance with the classification provided by Suraji et al. [88]. The aggregation of these data by means of the k-means cluster analysis allowed the definition of the role of each accident determinant and its variables in the most probable accident causation, generating a set of critical information for the definition of the profile of each accident in a teleological manner, i.e., providing information on both the system′s risk factors and those related to legal responsibility. 

The Matrix of Descriptors was used to synthesize data extracted from the ESAW reports into Boolean vectors, making available a basis for their systemic classification based on the accident descriptors and sub-descriptors. Therefore, MoD should be considered as a supportive tool for synthesizing information from the ESAW accident reports, providing a dataset that guarantees the logical disjunction of the accidents′ sub-descriptors. In practice, qualitative information contained in the reports is translated into a simply set of Boolean vectors (one vector for each accident), where the analyst inputs “1” if from the report emerges that a certain aspect affected the occurrence of the accident (e.g., if the electric accident occurred to a bricklayer while fixing a wall, the x_21_ variable is equal to 1). In this way, it is easier for the analyst to capture information from the ESAW reports, even from those ones filled in an improper manner: in our case study, 97 reports of accidents out of 116 could be used. Such a transformation of categorical variables into dichotomous variables for k-means cluster analysis allows for easy interpretation [89,90]. The results achieved at the end of each step of the proposed procedure were statistically verified by means of the ANOVA test and an excerpt of this is provided in the Supplementary Materials.

Overall, the merit of the proposed approach relies on the possibility of processing ESAW reports in an objective manner, eliciting valuable information concerning both distal and proximal factors, which can be used both by safety managers and decision makers to trace specific risk profiles. In other words, the combination of the MoD filtering and the aggregation through the cluster analysis can provide practical suggestions of where and how to act in order to reduce the repetition of similar accidents. Such an approach accomplishes other studies that have dealt with the management of ESAW data by means of cluster analysis [31,32]. However, while the latter consider any kind of accident occurred in a certain type of industry providing a wide perspective on it, our study is focused on the analysis of a specific type of accident to effectively draw up an accident scenario to be used to determine specific risk profiles, consistently with [30]. In addition, it has to point out that the proposed methodology allows a more thorough characterization of the risk profile, including both the system′s risk characteristics and legal responsibilities, which are key-factors in risk profiling and modeling in the construction industry [81]. This can reduce the distance between theoretical issues and practical needs of companies, in line with the research clues addressed among others by [91,92]. As far as the k-means cluster analysis is concerned, the procedure implemented in this study relies on its application twice: such an approach allows for a better definition of the centroids, guaranteeing a clearer evaluation of the mutual relationships among the accident′s variables, in line with research cues by Swuste et al. [93]. Furthermore, such an approach can reduce the limitations of k-means clustering due to its sensitiveness sensitive to noise and outlier points [52]. 

All things considered, the proposed approach can support safety managers in the preliminary characterization of risk scenarios [94,95], acting preventively and proactively to augment the effectiveness of OHS measures. The methodology can be applied for analyzing accidents statistics provided in accordance with the ESAW rules. Hence, its usability in different contexts and different countries (e.g., EU member states) is facilitated. Accordingly, this study can be valuable for theory development as well as for practitioners and decision makers. 

### 4.3. Limitations

The main limitation of the proposed approach is due to the complexity of calculations, requiring specific skills and training in statistical analysis. At the same time, a further drawback of the proposed approach relies on the ESAW coding system itself. As remarked by Jacinto et al. [34], the skills of coders have a substantial impact on the coding reliability. Hence, the more the coding quality can be guaranteed, the more the proposed approach can provide effective results. 

In addition, we have to remark that in this study the predictive role played by the results of our approach is limited to a “static” character if compared to the other approaches and models proposed in literature (e.g., [96,97]), which have a “dynamic” nature.

## 5. Conclusions

Learning from accidents is considered a fundamental step forward to guarantee a more generic prevention of their repetition [98]. The present study aimed at providing a methodology for capturing information from accidents databases developed following the ESAW protocol. For this purpose, a procedure for transforming categorical information into dichotomous variables was developed for filtering reports data, which can be further processed by means of the k-means cluster analysis. According to the proposed approach, this tool is used twice for a better definition of the centroids, guaranteeing a clearer evaluation of the mutual relationships among the accident′s variables. Such an approach allows the definition of both distal and proximal characteristics of a specific accident type, showing the role of each accident determinant and its variables in the most probable accident causation modes. Consequently, risk profiles based on the relationships existing among the accident determinants and the accident typical modes can be defined, providing practical information for the implementation of ad hoc safety measures. 

The output of the study can be considered more relevant and thus able to enhance research in this specific field of occupational safety when considering the case study context, i.e., the electrical accidents in the construction industry. The achieved results can be used to reduce the occurrence of similar accidents by means of specific OHS measures in this sector. They also offer a basis for a wider application of the proposed methodology to different accident types. 

Hence, additional applications of such an approach are expected in order to better validate the procedure augmenting knowledge on risk profiling from accidents databases, also considering different typologies of risks both in the construction industry as well as in other sectors.

## Figures and Tables

**Table 1:** Results of the statistical analysis of variables (descriptive statistics).

Variables	*N*	Minimum	Maximum	Mean		Deviat.	Variance
	Statistics	Statistics	Statistics	Statistics	Stand. Error	Statistics	Statistics
x_11_	97	0.000	1.000	0.02062	0.014503	0.142842	0.20
x_12_	97	0.000	1.000	0.10309	0.031035	0.305660	0.093
x_13_	97	0.000	1.000	0.56701	0.050571	0.498063	0.248
x_14_	97	0.000	1.000	0.30928	0.047173	0.464597	0.216
x_21_	97	0.000	1.000	0.67010	0.047987	0.472618	0.223
x_22_	97	0.000	1.000	0.29897	0.046725	0.460184	0.212
x_23_	97	0.000	1.000	0.03093	0.017669	0.174022	0.030
x_31_	97	0.000	1.000	0.47423	0.050963	0.501929	0.252
x_32_	97	0.000	1.000	0.32990	0.047987	0.472618	0.223
x_33_	97	0.000	1.000	0.18557	0.039677	0.390776	0.153
x_34_	97	0.000	1.000	0.01031	0.010309	0.101535	0.010
x_41_	97	0.000	1.000	0.82474	0.038803	0.382162	0.146
x_42_	97	0.000	1.000	0.17526	0.038803	0.382162	0.146
Valid (listwise)	97						

## Supplementary Materials

Available online at https://www.mdpi.com/1660-4601/16/23/4748/s1.
