# Supplementary material for: Risk Profiling from the European Statistics on Accidents at Work (ESAW) Accidents′ Databases: A Case Study in Construction Sites
Source: Int J Environ Res Public Health. 2019 Nov 27;16(23):4748. doi: 10.3390/ijerph16234748 (PMC6926752; doi:10.3390/ijerph16234748)
Supplement: Supplementary file 1 [file ijerph-16-04748-s001.zip › ijerph-650211_Supplementary material - ANOVA EXCERPTS.pdf]

Your trial period for SPSS for Windows will expire in 15867 days.

```
GET
  FILE='C:\Documents and Settings\Giuliano\Documenti\Downloads\elettrocuzio'+
 'ne_costruzioni.sav'.
DATASET NAME InsiemeDati1 WINDOW=FRONT.
QUICK CLUSTER
  IMP_cantiere IMP_opera IMP_distribuzione IMP_macchinario EDILE
  IMPIANTISTICA IMPREVISTA att_infortunato att_lavoratori
  estesa_responsabile solo_responsabile A_NORMA NON_A_NORMA
  /MISSING=LISTWISE
  /CRITERIA= CLUSTER(2) MXITER(10) CONVERGE(0)
  /METHOD=KMEANS(NOUPDATE)
  /PRINT INITIAL.
```

Note

Output creato		22-OCT-2019 15:29:58
Commenti		
Input	Dati	C:\Documents and Settings\Giuliano\Documenti\Downloads\elettrocuzione_costruzioni.sav
	File di dati attivo	InsiemeDati1
	Filtro	<nessuno>
	Peso	<nessuno>
	Distingui	<nessuno>
	N. di righe nel file dati di lavoro	97
Gestione valori mancanti	Definizione di valore mancante	I valori mancanti definiti dall'utente vengono considerati mancanti.
	Casi utilizzati	Le statistiche sono basate sui casi che non hanno valori mancanti per qualsiasi variabile di raggruppamento utilizzata.
Sintassi		QUICK CLUSTER IMP_cantiere IMP_opera IMP_distribuzione IMP_macchinario EDILE IMPIANTISTICA IMPREVISTA att_infortunato att_lavoratori estesa_responsabile solo_responsabile A_NORMA NON_A_NORMA /MISSING=LISTWISE /CRITERIA= CLUSTER(2) MXITER(10) CONVERGE(0) /METHOD=KMEANS(NOUPDATE) /PRINT INITIAL.
Risorse	Tempo del processore	0:00:00,00
	Tempo trascorso	0:00:00,00
	Area di lavoro richiesta	1920 byte

[InsiemeDati1] C:\Documents and Settings\Giuliano\Documenti\Downloads
\elettrocuzione_costruzioni.sav

Centri dei cluster iniziali

	Cluster	
	1	2
IMP_cantiere	,000	,000
IMP_opera	1,000	,000
IMP_distribuzione	,000	,000
IMP_macchinario	,000	1,000
EDILE	,000	,000
IMPIANTISTICA	,000	1,000
IMPREVISTA	1,000	,000
att_infortunato	,000	1,000
att_lavoratori	1,000	,000
estesa_responsabile	,000	,000
solo_responsabile	,000	,000
A_NORMA	1,000	,000
NON_A_NORMA	,000	1,000

Quick Cluster

Variables names	codes
IMP_cantiere	X_{11}
IMP_opera	X_{12}
IMP_distribuzione	X_{13}
IMP_macchinario	X_{14}
EDILE	X_{21}
IMPIANTISTICA	X_{22}
IMPREVISTA	X_{23}
att_infortunato	X_{31}
att_lavoratori	X_{32}
estesa_responsabile	X_{33}
solo_responsabile	X_{34}
A_NORMA	X_{41}
NON_A_NORMA	X_{42}

Cronologia iterazioni[a]

Iterazione	Modifiche ai centri dei cluster	
	1	2
1	1,923	,902
2	,172	,319
3	,030	,065
4	,000	,000

a. Convergenza ottenuta poiché nei centri dei cluster non sono presenti modifiche o sono presenti modifiche minime. La variazione massima assoluta delle coordinate per qualsiasi centro è ,000. L'iterazione corrente è 4. La distanza minima tra i centri iniziali è 2,828.

Centri dei cluster finali

	Cluster	
	1	2
IMP_cantiere	,031	,000
IMP_opera	,123	,063
IMP_distribuzione	,846	,000
IMP_macchinario	,000	,938
EDILE	,985	,031
IMPIANTISTICA	,000	,906
IMPREVISTA	,015	,063
att_infortunato	,385	,656
att_lavoratori	,369	,250
estesa_responsabile	,231	,094
solo_responsabile	,015	,000
A_NORMA	,892	,688
NON_A_NORMA	,108	,313

Numero di casi in ogni cluster

Cluster	1	65,000
	2	32,000
Validi		97,000
Mancanti		,000

```
* Convalida i dati.
VALIDATEDATA
  VARIABLES=IMP_cantiere IMP_opera IMP_distribuzione IMP_macchinario EDILE
  IMPIANTISTICA IMPREVISTA att_infortunato att_lavoratori
  estesa_responsabile solo_responsabile A_NORMA NON_A_NORMA
 /VARCHECKS STATUS=ON PCTMISSING=70 PCTEQUAL=95 PCTUNEQUAL=90
 /CASECHECKS REPORTEMPTY=YES SCOPE=ALLVARS
 /CASEREPORT DISPLAY=YES MINVIOLATIONS=1 CASELIMIT=FIRSTN(100).
```

Convalida dati

Note

Output creato		22-OCT-2019 15:32:47
Commenti		
Input	Dati	C:\Documents and Settings\Giuliano\Documenti\Downloads\elettrocuzione_costruzioni.sav
	File di dati attivo	InsiemeDati1
	Filtro	<nessuno>
	Peso	<nessuno>
	Distingui	<nessuno>
	N. di righe nel file dati di lavoro	97
Sintassi		VALIDATEDATA VARIABLES=IMP_cantiere IMP_opera IMP_distribuzione IMP_macchinario EDILE IMPIANTISTICA IMPREVISTA att_infortunato att_lavoratori estesa_responsabile solo_responsabile A_NORMA NON_A_NORMA /VARCHECKS STATUS=ON PCTMISSING=70 PCTEQUAL=95 PCTUNEQUAL=90 /CASECHECKS REPORTEMPTY=YES SCOPE=ALLVARS /CASEREPORT DISPLAY=YES MINVIOLATIONS=1 CASELIMIT=FIRSTN(100).
Risorse	Tempo del processore	0:00:00,05
	Tempo trascorso	0:00:00,05

```
[InsiemeDati1] C:\Documents and Settings\Giuliano\Documenti\Downloads
\elettrocuzione_costruzioni.sav
```

Avvisi

Alcuni o tutti i risultati richiesti non verranno visualizzati perché tutti i casi, le variabili o i valori dei dati hanno soddisfatto i controlli richiesti.

Controlli delle variabili

Categorica	Casi costanti > 95	IMP_cantiere<br>IMPREVISTA<br>solo_responsabile

Ciascuna variabile viene segnalata per ogni controllo non superato

```
QUICK CLUSTER
  IMP_cantiere IMP_opera IMP_distribuzione IMP_macchinario EDILE
  IMPIANTISTICA IMPREVISTA att_infortunato att_lavoratori
  estesa_responsabile solo_responsabile A_NORMA NON_A_NORMA
  /MISSING=LISTWISE
  /CRITERIA= CLUSTER(2) MXITER(10) CONVERGE(0)
  /METHOD=KMEANS(NOUPDATE)
  /SAVE CLUSTER DISTANCE
  /PRINT INITIAL ANOVA CLUSTER DISTAN.
```

Quick Cluster

Note

Output creato		22-OCT-2019 15:51:49
Commenti		
Input	Dati	C:\Documents and Settings\Giuliano\Documenti\Downloads\elettrocuzione_costruzioni.sav
	File di dati attivo	InsiemeDati1
	Filtro	<nessuno>
	Peso	<nessuno>
	Distingui	<nessuno>
	N. di righe nel file dati di lavoro	97
Gestione valori mancanti	Definizione di valore mancante	I valori mancanti definiti dall'utente vengono considerati mancanti.
	Casi utilizzati	Le statistiche sono basate sui casi che non hanno valori mancanti per qualsiasi variabile di raggruppamento utilizzata.
Sintassi		QUICK CLUSTER IMP_cantiere IMP_opera IMP_distribuzione IMP_macchinario EDILE IMPIANTISTICA IMPREVISTA att_infortunato att_lavoratori estesa_responsabile solo_responsabile A_NORMA NON_A_NORMA /MISSING=LISTWISE /CRITERIA= CLUSTER(2) MXITER(10) CONVERGE(0) /METHOD=KMEANS(NOUPDATE) /SAVE CLUSTER DISTANCE /PRINT INITIAL ANOVA CLUSTER DISTAN.
Risorse	Tempo del processore	0:00:00,03
	Tempo trascorso	0:00:00,05
	Area di lavoro richiesta	2232 byte
Variabili create o modificate	QCL_1	Numero di cluster del caso
	QCL_2	Distanza del caso dal centro di classificazione dei cluster

```
[InsiemeDati1] C:\Documents and Settings\Giuliano\Documenti\Downloads
\elettrocuzione_costruzioni.sav
```

Centri dei cluster iniziali

	Cluster	
	1	2
IMP_cantiere	,000	,000
IMP_opera	1,000	,000
IMP_distribuzione	,000	,000
IMP_macchinario	,000	1,000
EDILE	,000	,000
IMPIANTISTICA	,000	1,000
IMPREVISTA	1,000	,000
att_infortunato	,000	1,000
att_lavoratori	1,000	,000
estesa_responsabile	,000	,000
solo_responsabile	,000	,000
A_NORMA	1,000	,000
NON_A_NORMA	,000	1,000

Cronologia iterazioni[a]

Iterazione	Modifiche ai centri dei cluster	
	1	2
1	1,923	,902
2	,172	,319
3	,030	,065
4	,000	,000

a. Convergenza ottenuta poiché nei centri dei cluster non sono presenti modifiche o sono presenti modifiche minime. La variazione massima assoluta delle coordinate per qualsiasi centro è ,000. L'iterazione corrente è 4. La distanza minima tra i centri iniziali è 2,828.

Cluster di appartenenza

Numero di caso	Cluster	Distanza
1	1	2,012
2	1	,969
3	2	1,075
4	1	1,910
5	1	,969
6	1	2,035
7	2	,637
8	2	1,403
9	1	,814
10	2	1,705
11	1	,969
12	1	,969
13	2	,637
14	1	,795
15	2	1,104
16	1	,795
17	1	,814
18	2	,637
19	1	,969
20	1	,814
21	1	,814
22	1	,795
23	1	,814
24	1	1,442
25	1	,814
26	1	,814
27	2	1,104
28	2	1,237
29	1	,814
30	1	,814
31	2	1,104
32	1	,814
33	1	,814
34	1	,814
35	1	,814
36	2	1,510
37	1	,814
38	1	,814
39	2	,637
40	1	2,094
41	1	1,442
42	1	,795
43	1	,795

Cluster di appartenenza

Numero di caso	Cluster	Distanza
44	1	,814
45	2	,637
46	2	1,961
47	1	1,442
48	1	1,442
49	2	,637
50	2	,637
51	1	,795
52	2	,637
53	2	,637
54	2	,637
55	2	1,104
56	1	,795
57	1	,795
58	1	,795
59	1	,814
60	1	,814
61	1	,795
62	1	2,035
63	1	,795
64	1	,795
65	2	1,686
66	2	1,403
67	2	1,403
68	1	1,918
69	1	,969
70	1	,795
71	2	1,928
72	1	,795
73	2	,637
74	1	,795
75	1	,795
76	1	,969
77	2	1,075
78	1	,814
79	2	1,237
80	2	,637
81	1	,969
82	1	,969
83	1	,795
84	2	1,075
85	2	,637
86	1	1,484
87	1	,969
88	1	,969
89	1	,814
90	2	,637
91	2	,637
92	1	1,484
93	1	,795
94	1	,969
95	1	,969
96	1	,814
97	1	,814

Centri dei cluster finali

	Cluster	
	1	2
IMP_cantiere	,031	,000
IMP_opera	,123	,063
IMP_distribuzione	,846	,000
IMP_macchinario	,000	,938
EDILE	,985	,031
IMPIANTISTICA	,000	,906
IMPREVISTA	,015	,063
att_infortunato	,385	,656
att_lavoratori	,369	,250
estesa_responsabile	,231	,094
solo_responsabile	,015	,000
A_NORMA	,892	,688
NON_A_NORMA	,108	,313

Distanze tra i centri dei cluster finali

Cluster	1	2
1		1,877
2	1,877	

ANOVA

	Cluster		Errore			
	Media dei quadrati	df	Media dei quadrati	df	F	Sig.
IMP_cantiere	,020	1	,020	95	,995	,321
IMP_opera	,079	1	,094	95	,841	,361
IMP_distribuzione	15,353	1	,089	95	172,371	,000
IMP_macchinario	18,847	1	,020	95	954,897	,000
EDILE	19,490	1	,021	95	947,874	,000
IMPIANTISTICA	17,611	1	,029	95	615,378	,000
IMPREVISTA	,048	1	,030	95	1,581	,212
att_infortunato	1,582	1	,238	95	6,650	,011
att_lavoratori	,305	1	,223	95	1,370	,245
estesa_responsabile	,403	1	,150	95	2,683	,105
solo_responsabile	,005	1	,010	95	,490	,486
A_NORMA	,899	1	,138	95	6,512	,012
NON_A_NORMA	,899	1	,138	95	6,512	,012

I test F devono essere utilizzati solo per motivi descrittivi poiché i cluster sono stati scelti per ottimizzare le differenze tra i casi in diversi cluster. I livelli di significatività osservati non sono perciò corretti e non possono quindi essere interpretati come test dell'ipotesi che le medie dei cluster siano uguali.

Numero di casi in ogni cluster

Cluster	1	65,000
	2	32,000
Validi		97,000
Mancanti		,000

```
QUICK CLUSTER
  IMP_cantiere IMP_opera IMP_distribuzione IMP_macchinario EDILE
  IMPIANTISTICA IMPREVISTA att_infortunato att_lavoratori
  estesa_responsabile solo_responsabile A_NORMA NON_A_NORMA
  /MISSING=LISTWISE
  /CRITERIA= CLUSTER(3) MXITER(10) CONVERGE(0)
```

```
/METHOD=KMEANS(NOUPDATE)
/SAVE CLUSTER DISTANCE
/PRINT INITIAL ANOVA CLUSTER DISTAN.
```

Quick Cluster

Note

Output creato		22-OCT-2019 16:22:06
Commenti		
Input	Dati	C:\Documents and Settings\Giuliano\Documenti\Downloads\elettrocuzione_costruzioni.sav
	File di dati attivo	InsiemeDati1
	Filtro	<nessuno>
	Peso	<nessuno>
	Distingui	<nessuno>
	N. di righe nel file dati di lavoro	97
Gestione valori mancanti	Definizione di valore mancante	I valori mancanti definiti dall'utente vengono considerati mancanti.
	Casi utilizzati	Le statistiche sono basate sui casi che non hanno valori mancanti per qualsiasi variabile di raggruppamento utilizzata.
Sintassi		QUICK CLUSTER IMP_cantiere IMP_opera IMP_distribuzione IMP_macchinario EDILE IMPIANTISTICA IMPREVISTA att_infortunato att_lavoratori estesa_responsabile solo_responsabile A_NORMA NON_A_NORMA /MISSING=LISTWISE /CRITERIA= CLUSTER(3) MXITER(10) CONVERGE(0) /METHOD=KMEANS(NOUPDATE) /SAVE CLUSTER DISTANCE /PRINT INITIAL ANOVA CLUSTER DISTAN.
Risorse	Tempo del processore	0:00:00,00
	Tempo trascorso	0:00:00,00
	Area di lavoro richiesta	2696 byte
Variabili create o modificate	QCL_3	Numero di cluster del caso
	QCL_4	Distanza del caso dal centro di classificazione dei cluster

```
[InsiemeDati1] C:\Documents and Settings\Giuliano\Documenti\Downloads
\elettrocuzione_costruzioni.sav
```

Centri dei cluster iniziali

	Cluster		
	1	2	3
IMP_cantiere	,000	,000	,000
IMP_opera	1,000	,000	,000
IMP_distribuzione	,000	1,000	,000
IMP_macchinario	,000	,000	1,000
EDILE	,000	1,000	,000
IMPIANTISTICA	,000	,000	1,000
IMPREVISTA	1,000	,000	,000
att_infortunato	,000	,000	1,000
att_lavoratori	1,000	,000	,000
estesa_responsabile	,000	1,000	,000
solo_responsabile	,000	,000	,000
A_NORMA	1,000	1,000	,000
NON_A_NORMA	,000	,000	1,000

Cronologia iterazioni[a]

	Modifiche ai centri dei cluster		
Iterazione	1	2	3
1	1,328	,883	,872
2	,703	,074	,277
3	,348	,076	,060
4	,208	,059	,000
5	,000	,000	,000

a. Convergenza ottenuta poiché nei centri dei cluster non sono presenti modifiche o sono presenti modifiche minime. La variazione massima assoluta delle coordinate per qualsiasi centro è ,000. L'iterazione corrente è 5. La distanza minima tra i centri iniziali è 2,449.

Cluster di appartenenza

Numero di caso	Cluster	Distanza
1	1	1,814
2	2	,926
3	3	1,113
4	1	,869
5	2	,926
6	1	1,660
7	3	,585
8	3	1,438
9	2	,719
10	1	1,491
11	2	,926
12	2	,926
13	3	,585
14	2	,818
15	3	1,082
16	2	,818
17	2	,719
18	3	,585
19	2	,926
20	2	,719
21	2	,719
22	2	,818
23	2	,719
24	1	1,075

Cluster di appartenenza

Numero di caso	Cluster	Distanza
25	2	,719
26	2	,719
27	3	1,082
28	3	1,202
29	2	,719
30	2	,719
31	3	1,082
32	2	,719
33	2	,719
34	2	,719
35	2	,719
36	3	1,531
37	2	,719
38	2	,719
39	3	,585
40	1	1,350
41	1	1,075
42	2	,818
43	2	,818
44	2	,719
45	3	,585
46	1	1,578
47	1	1,075
48	1	1,075
49	3	,585
50	3	,585
51	2	,818
52	3	,585
53	3	,585
54	3	,585
55	3	1,082
56	2	,818
57	2	,818
58	2	,818
59	2	,719
60	2	,719
61	2	,818
62	1	1,660
63	2	,818
64	2	,818
65	3	1,761
66	3	1,438
67	3	1,438
68	1	1,247
69	2	,926
70	2	,818
71	1	1,660
72	2	,818
73	3	,585
74	2	,818
75	2	,818
76	2	,926
77	3	1,113
78	2	,719
79	3	1,202
80	3	,585

Cluster di appartenenza

Numero di caso	Cluster	Distanza
81	2	,926
82	2	,926
83	2	,818
84	3	1,113
85	3	,585
86	1	1,350
87	2	,926
88	2	,926
89	2	,719
90	3	,585
91	3	,585
92	1	1,350
93	2	,818
94	2	,926
95	2	,926
96	2	,719
97	2	,719

Centri dei cluster finali

	Cluster		
	1	2	3
IMP_cantiere	,133	,000	,000
IMP_opera	,667	,000	,000
IMP_distribuzione	,133	1,000	,000
IMP_macchinario	,067	,000	1,000
EDILE	,800	1,000	,000
IMPIANTISTICA	,067	,000	,966
IMPREVISTA	,133	,000	,034
att_infortunato	,600	,340	,655
att_lavoratori	,200	,415	,241
estesa_responsabile	,133	,245	,103
solo_responsabile	,067	,000	,000
A_NORMA	,400	1,000	,724
NON_A_NORMA	,600	,000	,276

Distanze tra i centri dei cluster finali

Cluster	1	2	3
1		1,460	1,740
2	1,460		2,058
3	1,740	2,058	

ANOVA

	Cluster		Errore			
	Media dei quadrati	df	Media dei quadrati	df	F	Sig.
IMP_cantiere	,113	2	,018	94	6,113	,003
IMP_opera	2,818	2	,035	94	79,464	,000
IMP_distribuzione	11,041	2	,018	94	598,738	,000
IMP_macchinario	9,894	2	,010	94	996,483	,000
EDILE	9,522	2	,026	94	372,931	,000
IMPIANTISTICA	9,216	2	,020	94	456,202	,000
IMPREVISTA	,104	2	,029	94	3,629	,030
att_infortunato	1,074	2	,234	94	4,579	,013
att_lavoratori	,433	2	,219	94	1,976	,144
estesa_responsabile	,213	2	,151	94	1,405	,250
solo_responsabile	,028	2	,010	94	2,838	,064
A_NORMA	2,314	2	,100	94	23,155	,000
NON_A_NORMA	2,314	2	,100	94	23,155	,000

I test F devono essere utilizzati solo per motivi descrittivi poiché i cluster sono stati scelti per ottimizzare le differenze tra i casi in diversi cluster. I livelli di significatività osservati non sono perciò corretti e non possono quindi essere interpretati come test dell'ipotesi che le medie dei cluster siano uguali.

Numero di casi in ogni cluster

Cluster	1	15,000
	2	53,000
	3	29,000
Validi		97,000
Mancanti		,000

```
QUICK CLUSTER
  IMP_cantiere IMP_opera IMP_distribuzione IMP_macchinario EDILE
  IMPIANTISTICA IMPREVISTA att_infortunato att_lavoratori
  estesa_responsabile solo_responsabile A_NORMA NON_A_NORMA
  /MISSING=LISTWISE
  /CRITERIA= CLUSTER(6) MXITER(10) CONVERGE(0)
  /METHOD=KMEANS(NOUPDATE)
  /SAVE CLUSTER DISTANCE
  /PRINT INITIAL ANOVA CLUSTER DISTAN.
```

Quick Cluster

Note

Output creato		22-OCT-2019 16:33:24
Commenti		
Input	Dati	C:\Documents and Settings\Giuliano\Documenti\ Downloads\elettrocuzione_ costruzioni.sav
	File di dati attivo	InsiemeDati1
	Filtro	<nessuno>
	Peso	<nessuno>
	Distingui	<nessuno>
	N. di righe nel file dati di lavoro	97
Gestione valori mancanti	Definizione di valore mancante	I valori mancanti definiti dall'utente vengono considerati mancanti.
	Casi utilizzati	Le statistiche sono basate sui casi che non hanno valori mancanti per qualsiasi variabile di raggruppamento utilizzata.
Sintassi		QUICK CLUSTER IMP_cantiere IMP_opera IMP_ distribuzione IMP_macchinario EDILE IMPIANTISTICA IMPREVISTA att_ infortunato att_lavoratori estesa_responsabile solo_ responsabile A_NORMA NON_A_ NORMA /MISSING=LISTWISE /CRITERIA= CLUSTER(6) MXITER(10) CONVERGE(0) /METHOD=KMEANS(NOUPDATE) /SAVE CLUSTER DISTANCE /PRINT INITIAL ANOVA CLUSTER DISTAN.
Risorse	Tempo del processore	0:00:00,00
	Tempo trascorso	0:00:00,00
	Area di lavoro richiesta	4136 byte
Variabili create o modificate	QCL_5	Numero di cluster del caso
	QCL_6	Distanza del caso dal centro di classificazione dei cluster

```
[InsiemeDati1] C:\Documents and Settings\Giuliano\Documenti\Downloads
\elettrocuzione_costruzioni.sav
```

Centri dei cluster iniziali

	Cluster					
	1	2	3	4	5	6
IMP_cantiere	,000	,000	,000	,000	,000	1,000
IMP_opera	1,000	,000	,000	1,000	,000	,000
IMP_distribuzione	,000	1,000	,000	,000	,000	,000
IMP_macchinario	,000	,000	1,000	,000	1,000	,000
EDILE	,000	1,000	,000	1,000	,000	1,000
IMPIANTISTICA	,000	,000	1,000	,000	1,000	,000
IMPREVISTA	1,000	,000	,000	,000	,000	,000
att_infortunato	,000	,000	,000	1,000	1,000	,000
att_lavoratori	1,000	,000	1,000	,000	,000	,000
estesa_responsabile	,000	1,000	,000	,000	,000	1,000
solo_responsabile	,000	,000	,000	,000	,000	,000
A_NORMA	1,000	1,000	,000	,000	1,000	,000
NON_A_NORMA	,000	,000	1,000	1,000	,000	1,000

Cronologia iterazioni[a]

Iterazione	Modifiche ai centri dei cluster					
	1	2	3	4	5	6
1	,707	,926	,688	,693	,166	,000
2	,000	,000	,000	,000	,000	,000

a. Convergenza ottenuta poiché nei centri dei cluster non sono presenti modifiche o sono presenti modifiche minime. La variazione massima assoluta delle coordinate per qualsiasi centro è ,000. L'iterazione corrente è 2. La distanza minima tra i centri iniziali è 2,000.

Cluster di appartenenza

Numero di caso	Cluster	Distanza
1	1	,707
2	2	,926
3	3	1,043
4	4	,693
5	2	,926
6	6	,000
7	5	,166
8	3	,688
9	2	,719
10	4	1,442
11	2	,926
12	2	,926
13	5	,166
14	2	,818
15	3	1,115
16	2	,818
17	2	,719
18	5	,166
19	2	,926
20	2	,719
21	2	,719
22	2	,818
23	2	,719
24	4	,938
25	2	,719
26	2	,719
27	3	1,115
28	5	1,248
29	2	,719
30	2	,719
31	3	1,115
32	2	,719
33	2	,719
34	2	,719
35	2	,719
36	3	1,245
37	2	,719
38	2	,719
39	5	,166
40	4	1,371
41	4	,938
42	2	,818
43	2	,818
44	2	,719
45	5	,166
46	1	,707

Cluster di appartenenza

Numero di caso	Cluster	Distanza
47	4	,938
48	4	,938
49	5	,166
50	5	,166
51	2	,818
52	5	,166
53	5	,166
54	5	,166
55	3	1,115
56	2	,818
57	2	,818
58	2	,818
59	2	,719
60	2	,719
61	2	,818
62	6	,000
63	2	,818
64	2	,818
65	3	1,621
66	3	,688
67	3	,688
68	4	1,371
69	2	,926
70	2	,818
71	3	1,418
72	2	,818
73	5	,166
74	2	,818
75	2	,818
76	2	,926
77	3	1,043
78	2	,719
79	5	1,248
80	5	,166
81	2	,926
82	2	,926
83	2	,818
84	3	1,043
85	5	,166
86	4	1,296
87	2	,926
88	2	,926
89	2	,719
90	5	,166
91	5	,166
92	4	1,296
93	2	,818
94	2	,926
95	2	,926
96	2	,719
97	2	,719

Centri dei cluster finali

	Cluster					
	1	2	3	4	5	6
IMP_cantiere	,000	,000	,000	,000	,000	1,000
IMP_opera	1,000	,000	,000	,800	,000	,000
IMP_distribuzione	,000	1,000	,000	,200	,000	,000
IMP_macchinario	,000	,000	1,000	,000	1,000	,000
EDILE	,000	1,000	,077	,900	,000	1,000
IMPIANTISTICA	,000	,000	,846	,100	1,000	,000
IMPREVISTA	1,000	,000	,077	,000	,000	,000
att_infortunato	,500	,340	,308	,800	,882	,000
att_lavoratori	,500	,415	,615	,100	,000	,000
estesa_responsabile	,000	,245	,077	,000	,118	1,000
solo_responsabile	,000	,000	,000	,100	,000	,000
A_NORMA	1,000	1,000	,308	,400	1,000	,000
NON_A_NORMA	,000	,000	,692	,600	,000	1,000

Distanze tra i centri dei cluster finali

Cluster	1	2	3	4	5	6
1		2,023	2,142	1,697	2,100	2,739
2	2,023		2,145	1,550	2,117	2,204
3	2,142	2,145		1,860	1,306	2,256
4	1,697	1,550	1,860		2,015	1,918
5	2,100	2,117	1,306	2,015		2,749
6	2,739	2,204	2,256	1,918	2,749	

ANOVA

	Cluster		Errore			
	Media dei quadrati	df	Media dei quadrati	df	F	Sig.
IMP_cantiere	,392	5	,000	91	.	.
IMP_opera	1,474	5	,018	91	83,823	,000
IMP_distribuzione	4,443	5	,018	91	252,689	,000
IMP_macchinario	4,144	5	,000	91	.	.
EDILE	3,924	5	,020	91	195,871	,000
IMPIANTISTICA	3,548	5	,028	91	124,532	,000
IMPREVISTA	,397	5	,010	91	39,121	,000
att_infortunato	1,133	5	,204	91	5,567	,000
att_lavoratori	,820	5	,191	91	4,301	,001
estesa_responsabile	,432	5	,137	91	3,146	,012
solo_responsabile	,018	5	,010	91	1,814	,118
A_NORMA	1,770	5	,057	91	31,164	,000
NON_A_NORMA	1,770	5	,057	91	31,164	,000

I test F devono essere utilizzati solo per motivi descrittivi poiché i cluster sono stati scelti per ottimizzare le differenze tra i casi in diversi cluster. I livelli di significatività osservati non sono perciò corretti e non possono quindi essere interpretati come test dell'ipotesi che le medie dei cluster siano uguali.

Numero di casi in ogni cluster

Cluster	1	2,000
	2	53,000
	3	13,000
	4	10,000
	5	17,000
	6	2,000
Validi		97,000
Mancanti		,000
