# Supplementary material for: Risk Profiling from the European Statistics on Accidents at Work (ESAW) Accidents′ Databases: A Case Study in Construction Sites
Source: Int J Environ Res Public Health. 2019 Nov 27;16(23):4748. doi: 10.3390/ijerph16234748 (PMC6926752; doi:10.3390/ijerph16234748)
Supplement: Supplementary file 1 [file ijerph-16-04748-s001.zip › ijerph-650211_Supplementary material - Distances Matrix_Statistics.pdf]

Variables names	codes
IMP_cantiere	x_{11}
IMP_opera	x_{12}
IMP_distribuzione	x_{13}
IMP_macchinario	x_{14}
EDILE	x_{21}
IMPIANTISTICA	x_{22}
IMPREVISTA	x_{23}
att_infortunato	x_{31}
att_lavoratori	x_{32}
estesa_responsabile	x_{33}
solo_responsabile	x_{34}
A_NORMA	x_{41}
NON_A_NORMA	x_{42}

```
GET
  FILE='C:\Documents and Settings\Giuliano\Desktop\elaborazioni cantieri 20'+
 '19.sav'.
DATASET NAME InsiemeDati1 WINDOW=FRONT.
DESCRIPTIVES
  VARIABLES=IMP_cantiere IMP_opera IMP_distribuzione IMP_macchinario EDILE
  IMPIANTISTICA IMPREVISTA att_infortunato att_lavoratori
  estesa_responsabile solo_responsabile A_NORMA NON_A_NORMA
  /STATISTICS=MEAN STDDEV VARIANCE MIN MAX SEMEAN .
```

Descriptive

[InsiemeDati1] C:\Documents and Settings\Giuliano\Desktop\elaborazioni cantieri 2019.sav

Descriptive Statistics

Variables	N	Minimum	Maximum	Mean		Deviat.	Variance
	Statistics	Statistics	Statistics	Statistics	Stand. Error	Statistics	Statistics
x_{11}	97	0.000	1.000	0.02062	0.014503	0.142842	0.20
x_{12}	97	0.000	1.000	0.10309	0.031035	0.305660	0.093
x_{13}	97	0.000	1.000	0.56701	0.050571	0.498063	0.248
x_{14}	97	0.000	1.000	0.30928	0.047173	0.464597	0.216
x_{21}	97	0.000	1.000	0.67010	0.047987	0.472618	0.223
x_{22}	97	0.000	1.000	0.29897	0.046725	0.460184	0.212
x_{23}	97	0.000	1.000	0.03093	0.017669	0.174022	0.030
x_{31}	97	0.000	1.000	0.47423	0.050963	0.501929	0.252
x_{32}	97	0.000	1.000	0.32990	0.047987	0.472618	0.223
x_{33}	97	0.000	1.000	0.18557	0.039677	0.390776	0.153
x_{34}	97	0.000	1.000	0.01031	0.010309	0.101535	0.010
x_{41}	97	0.000	1.000	0.82474	0.038803	0.382162	0.146
x_{42}	97	0.000	1.000	0.17526	0.038803	0.382162	0.146
Valid (listwise)	97						

Statistiche descrittive

	N	Minimo	Massimo	Media		Deviazion	Varianza
	Statistica	Statistica	Statistica	Statistica	Errore std	Statistica	Statistica
IMP_cantiere	97	,000	1,000	,02062	,014503	,142842	,020
IMP_opera	97	,000	1,000	,10309	,031035	,305660	,093
IMP_distribuzione	97	,000	1,000	,56701	,050571	,498063	,248
IMP_macchinario	97	,000	1,000	,30928	,047173	,464597	,216
EDILE	97	,000	1,000	,67010	,047987	,472618	,223
IMPIANTISTICA	97	,000	1,000	,29897	,046725	,460184	,212
IMPREVISTA	97	,000	1,000	,03093	,017669	,174022	,030
att_infortunato	97	,000	1,000	,47423	,050963	,501929	,252
att_lavoratori	97	,000	1,000	,32990	,047987	,472618	,223
estesa_responsabile	97	,000	1,000	,18557	,039677	,390776	,153
solo_responsabile	97	,000	1,000	,01031	,010309	,101535	,010
A_NORMA	97	,000	1,000	,82474	,038803	,382162	,146
NON_A_NORMA	97	,000	1,000	,17526	,038803	,382162	,146
Validi (listwise)	97						

Distances

```
[InsiemeDati1] C:\Documents and Settings\Giuliano\Desktop\elaborazioni cantieri 2019.sav
```

Riepilogo dei casi

Casi							
Validi		Rifiutati				Totale	
		Valore mancante		Valore binario fuori intervallo[a]			
N	Percentuale	N	Percentuale	N	Percentuale	N	Percentuale
97	100,0%	0	,0%	0	,0%	97	100,0%

a. Valore diverso sia da 1 che da 0.

Distances Matrix
[Binary Lance-and-Williams nonmetric measure]

Matrice delle distanze

	Misura non metrica Lance e Williams binaria												
	IMP_cantiere	IMP_opera	IMP_ distribuzione	IMP_ macchinario	EDILE	IMPIANTI STICA	IMPREVISTA	att_infortunato	att_lavoratori	estesa_ responsabile	solo_ responsabile	A_NORMA	NON_A_ NORMA
IMP_cantiere	,000	1,000	1,000	1,000	,940	1,000	1,000	1,000	1,000	,800	1,000	1,000	,789
IMP_opera	1,000	,000	1,000	1,000	,813	,949	,692	,750	,905	1,000	,818	,867	,704
IMP_distribuzione	1,000	1,000	,000	1,000	,083	1,000	1,000	,604	,494	,644	1,000	,215	,944
IMP_macchinario	1,000	1,000	1,000	,000	,979	,051	,939	,500	,742	,875	1,000	,618	,617
EDILE	,940	,813	,083	,979	,000	1,000	1,000	,550	,505	,639	,970	,214	,805
IMPIANTISTICA	1,000	,949	1,000	,051	1,000	,000	1,000	,493	,770	,872	1,000	,615	,652
IMPREVISTA	1,000	,692	1,000	,939	1,000	1,000	,000	,918	,943	1,000	1,000	,952	,900
att_infortunato	1,000	,750	,604	,500	,550	,493	,918	,000	1,000	1,000	1,000	,397	,746
att_lavoratori	1,000	,905	,494	,742	,505	,770	,943	1,000	,000	1,000	1,000	,518	,796
estesa_responsabile	,800	1,000	,644	,875	,639	,872	1,000	1,000	1,000	,000	1,000	,694	,829
solo_responsabile	1,000	,818	1,000	1,000	,970	1,000	1,000	1,000	1,000	1,000	,000	1,000	,889
A_NORMA	1,000	,867	,215	,618	,214	,615	,952	,397	,518	,694	1,000	,000	1,000
NON_A_NORMA	,789	,704	,944	,617	,805	,652	,900	,746	,796	,829	,889	1,000	,000

Questa è una matrice di dissimilarità
