# Supplementary material for: Risk Profiling from the European Statistics on Accidents at Work (ESAW) Accidents′ Databases: A Case Study in Construction Sites
Source: Int J Environ Res Public Health. 2019 Nov 27;16(23):4748. doi: 10.3390/ijerph16234748 (PMC6926752; doi:10.3390/ijerph16234748)
Supplement: Supplementary file 1 [file ijerph-16-04748-s001.zip › ijerph-650211_Supplementary material -4 cluster without centroids.pdf]

4-Cluster solution without centroids

Centri dei cluster iniziali

	Cluster			
	1	2	3	4
IMP_cantiere	,000	,000	,000	,000
IMP_opera	1,000	,000	,000	1,000
IMP_distribuzione	,000	1,000	,000	,000
IMP_macchinario	,000	,000	1,000	,000
EDILE	,000	1,000	,000	1,000
IMPIANTISTICA	,000	,000	1,000	,000
IMPREVISTA	1,000	,000	,000	,000
att_infortunato	,000	,000	1,000	1,000
att_lavoratori	1,000	,000	,000	,000
estesa_responsabile	,000	1,000	,000	,000
solo_responsabile	,000	,000	,000	,000
A_NORMA	1,000	1,000	1,000	,000
NON_A_NORMA	,000	,000	,000	1,000

Cronologia iterazioni[a]

	Modifiche ai centri dei cluster			
Iterazione	1	2	3	4
1	,707	,895	,585	,716
2	,000	,080	,000	,285
3	,000	,000	,000	,000

a. Convergenza ottenuta poiché nei centri dei cluster non sono presenti modifiche o sono presenti modifiche minime. La variazione massima assoluta delle coordinate per qualsiasi centro è ,000. L'iterazione corrente è 3. La distanza minima tra i centri iniziali è 2,449.

Centri dei cluster finali

	Cluster			
	1	2	3	4
IMP_cantiere	,000	,000	,000	,154
IMP_opera	1,000	,000	,000	,615
IMP_distribuzione	,000	1,000	,000	,154
IMP_macchinario	,000	,000	1,000	,077
EDILE	,000	1,000	,000	,923
IMPIANTISTICA	,000	,000	,966	,077
IMPREVISTA	1,000	,000	,034	,000
att_infortunato	,500	,340	,655	,615
att_lavoratori	,500	,415	,241	,154
estesa_responsabile	,000	,245	,103	,154
solo_responsabile	,000	,000	,000	,077
A_NORMA	1,000	1,000	,724	,308
NON_A_NORMA	,000	,000	,276	,692

ANOVA

	Cluster		Errore			
	Media dei quadrati	df	Media dei quadrati	df	F	Sig.
IMP_cantiere	,089	3	,018	93	4,881	,003
IMP_opera	1,964	3	,033	93	59,363	,000
IMP_distribuzione	7,374	3	,018	93	405,237	,000
IMP_macchinario	6,600	3	,010	93	664,902	,000
EDILE	6,840	3	,010	93	689,137	,000
IMPIANTISTICA	6,147	3	,020	93	302,702	,000
IMPREVISTA	,647	3	,010	93	62,342	,000
att_infortunato	,723	3	,237	93	3,056	,032
att_lavoratori	,358	3	,219	93	1,632	,187
estesa_responsabile	,156	3	,153	93	1,019	,388
solo_responsabile	,022	3	,010	93	2,237	,089
A_NORMA	1,819	3	,092	93	19,762	,000
NON_A_NORMA	1,819	3	,092	93	19,762	,000

I test F devono essere utilizzati solo per motivi descrittivi poiché i cluster sono stati scelti per ottimizzare le differenze tra i casi in diversi cluster. I livelli di significatività osservati non sono perciò corretti e non possono quindi essere interpretati come test dell'ipotesi che le medie dei cluster siano uguali.

Numero di casi in ogni cluster

Cluster	1	2,000
	2	53,000
	3	29,000
	4	13,000
Validi		97,000
Mancanti		,000
